# (+)-catechin protects PC12 cells against CORT-induced oxidative stress and pyroptosis through the pathways of PI3K/AKT and Nrf2/HO-1/NF-κB

**DOI:** 10.3389/fphar.2024.1450211

**Published:** 2024-08-28

**Authors:** Lai Chencen, Zhang Shuo, Chen Zhiyu, Fu Xiaoyu, Zhang Min, Wang Pengjiao, Gao Xiuli

**Affiliations:** ^1^ State Key Laboratory of Functions and Applications of Medicinal Plants and School of Pharmacy, Guizhou Medical University, Guiyang, China; ^2^ Center of Microbiology and Biochemical Pharmaceutical Engineering, Guizhou Medical University, Guiyang, China; ^3^ Department of Nosocomial Infection, The First Affiliated Hospital of Guizhou University of Traditional Chinese Medicine, Guiyang, China; ^4^ Experimental Animal Center of Guizhou Medical University, Guiyang, China; ^5^ Guizhou Provincial Engineering Research Center of Food Nutrition and Health, Guizhou Medical University, Guiyang, China

**Keywords:** (+)-catechin, oxidative stress, pyroptosis, PI3K/Akt signal pathway, Nrf2/HO-1/NF-κB signal pathway

## Abstract

Pyroptosis induced by oxidative stress is a significant contributor to mental health disorders, including depression (+)-Catechin (CA), a polyphenolic compound prevalent in various food sources, has been substantiated by prior research to exhibit potent antioxidant properties and potential antidepressant effects. Nonetheless, the precise antidepressive mechanisms and effects of CA remain incompletely elucidated. In this study, we employed corticosterone (CORT) and PC12 cells to develop a cellular model of depression, aiming to investigate the protective effects of CA against CORT-induced cellular damage. Our objective was to elucidate the underlying mechanisms of protective action. We utilized transcriptomic analysis to identify differentially expressed genes and employed bioinformatics approaches to predict the potential mechanisms of CA’s protective effects in PC12 cells. These transcriptomic predictions were subsequently validated through western blot analysis. The findings indicated that CA possesses the capacity to mitigate oxidative stress and suppress pyroptosis in PC12 cells via the activation of the PI3K/AKT signaling pathway. This activation subsequently modulates the Nrf2/HO1/NF-κB pathways, thereby providing protection to PC12 cells against damage induced by CORT. Furthermore, we investigated the interaction between CA and the Keap1 protein employing molecular docking and protein thermal shift assays. We propose that CA can activate Nrf2 through two mechanisms to decrease reactive oxygen species (ROS) levels and inhibit pyroptosis: one mechanism involves the activation of the PI3K/AKT signaling pathway, and the other involves direct binding to Keap1, leading to an increase in p-Nrf2.

## Introduction

Depression, a prevalent mental illness with serious consequences, has become one of the most significant mental health disorders worldwide in the 21st century (Rehm and Shield, 2019). Current estimates indicate that approximately 10% of the global population is affected by depression (Rao et al., 2021). With the accelerating pace of modern life and the rise in stress levels, the number of individuals suffering from depression is projected to increase. Particularly following the outbreak of Coronavirus Disease 2019 (COVID-19), stressors such as economic pressure and restricted activities have further exacerbated the incidence of various mental illnesses, including depression (Zhu et al., 2024). Depression has now become the second leading cause of death after cancer and has been recognized by the WHO as one of the main causes of disability worldwide. Currently, the commonly used antidepressants are mainly tricyclic antidepressants and monoamine oxidase inhibitors, but in clinical practice, about one-third of patients do not benefit from these two types of drug treatments (Nikolin et al., 2022). Furthermore, the limitations of those antidepressants, such as extended treatment durations and severe side effects (Hao et al., 2021), highlight the need of discovering more effective and less harmful alternatives for treatment.

The etiology of depression is multifaceted, encompassing strong associations with stress, inflammation, immune function, neurotransmitter imbalances, and genetic predispositions (Wang et al., 2022; Wang et al., 2021; McGuffin and Rivera, 2015; Uzbekov and Maximova, 2015). Oxidative stress, arising from an imbalance between pro-oxidant and antioxidant processes within the body, is pivotal in the development of depression (Bhatt et al., 2020). Prolonged exposure to oxidative stress may result in an overabundance of reactive oxygen species (ROS) in cells, leading to neurotoxic effects (Liao et al., 2023). During the development of depression, excessive accumulation of ROS in the brain can impair the antioxidant defense system of nerve cells, alter and damage the permeability of the cell and organelle membranes, leading to intracellular lipid peroxidation and organelle damage (Qin et al., 2007; He et al., 2024). Moreover, evidence indicates that ROS act as a key activator of the NLRP3 inflammasome (Liu et al., 2023), and a connection between depression and pyroptosis mediated by the NLRP3 inflammasome was reported recently (Xia et al., 2023). Pyroptosis is a form of programmed cell death. Unlike apoptosis, pyroptosis results in a pro-inflammatory outcome. Pyroptosis is initiated by activation of the inflammasome, particularly NLRP3, which plays a key role in neuroinflammation. Upon activation, NLRP3 triggers Caspase-1, leading to the maturation of the inflammatory cytokine IL-1β and the executioner of pyroptosis, GSDMD (Xie et al., 2019). This process culminates in cell death and the production of various pro-inflammatory mediators. Notably, elevated oxidative stress and pro-inflammatory mediators are prominently observed in the brains of patients with depression. Therefore, reducing oxidative stress and pro-inflammatory mediators to protect neurons from damage may be crucial for the treatment of depression (Xia et al., 2023). Modulating the NLRP3 inflammasome to diminish pyroptosis is essential for effective depression management.

Nuclear factor-like 2 (Nrf2) functions as a transcription factor that mitigates oxidative damage by initiating a suite of antioxidant response pathways. The interaction between Keap1 and Nrf2, wherein Keap1 typically inhibits the Nrf2 activity, is a classical regulatory mechanism (Baird and Yamamoto, 2020). After dissociation from Keap1, Nrf2 translocates to the nucleus and enhances the expression of antioxidant genes containing antioxidant response elements (ARE), such as heme oxygenase-1 (HO-1) and superoxide dismutase (SOD), which aid in the elimination of ROS and play a key role in maintaining cellular redox balance (Satoh et al., 2006). Beyond the classical regulatory mechanisms, Nrf2 activity is also modulated through Keap1-independent regulation. Kinases such as c-Jun N-terminal Kinase (JNK), Protein Kinase C (PKC) and Extracellular Signal-regulated Kinases (ERK) can promote the phosphorylation and nuclear translocation of Nrf2 (Zuo et al., 2022). Numerous studies have demonstrated that enhancing the phosphorylation and nuclear translocation of Nrf2 can ameliorate the depressive models both *in vivo* and *in vitro* (Chou et al., 2023; Sun et al., 2022; Si et al., 2023). Thus, augmenting the phosphorylation and nuclear translocation of Nrf2 to activate the cell’s intrinsic antioxidant system may represent a novel direction in the development of antidepressant drugs.

Catechins is a class of flavanol compounds characterized by multiple phenolic hydroxyl groups. These compounds are widely distributed in plants and are particularly abundant in certain fruits and edible plants such as cocoa, tea leaves, and raspberries. Catechins include a range of compounds such as (+)-catechin (CA), (−)-epicatechin (EC), (−)-epigallocatechin (EGC), (−)-epicatechingallate (ECG), and (−)-epigallocatechingallate (EGCG) (Bernatoniene and Kopustinskiene, 2018). Due to their potent antioxidant activity, catechins are recognized as important dietary supplements in countries including the United States, France, and Finland. (Wu et al., 2021). As one of the catechins, CA also exhibits significant antioxidant properties. The mechanism is likely similar to that of other catechins, involving the binding of free radicals through the phenolic hydroxyl groups present in its structure. This binding inhibits the chain reactions of free radicals and decreases lipid peroxidation within cells (Bernatoniene and Kopustinskiene, 2018). Known for its potent antioxidant properties, CA offers protective effects against various conditions, including liver and cardiac damage, DNA damage, and diabetes (Baranwal et al., 2022; Abd El-Aziz et al., 2012; Haza and Morales, 2011; Kobayashi et al., 2010). In terms of neuroprotection, CA has shown efficacy. A study by Margarita et al. demonstrated that CA, when combined with exercise, synergistically enhances neuroprotection in aging rats (Ramis et al., 2021). Heo et al. found that CA could mitigate Aβ-induced apoptosis (Heo and Lee, 2005). Additionally, studies have indicated that prolonged intake of CA can alleviate depressive behaviors induced by CORT in a rat model (Lee et al., 2013). However, this study primarily examines the mitigate effect of CA on the hypothalamic-pituitary-adrenal (HPA) axis and depressive-like behavior in rats, without elucidating the specific molecular mechanisms underlying CA’s antidepressant action. Given the high lipid content in the brain and the low antioxidant capacity of neurons, it is hypothesized that the antioxidant properties of CA is closely related to its antidepressant effect. However, the precise molecular mechanisms that connect the antioxidative and antidepressive effects of CA have yet to be comprehensively elucidated. In this study, PC12 cells with neuronal characteristics will be employed, and a concentration of 400 μM CORT will be used to establish a cellular model of depression, based on existing literature, to investigate the potential antidepressant mechanisms of CA.

## Materials and methods

### Antibodies and reagents

(+)-Catechin (purity > 97%) was sourced from Macklin in China. Aladdin, also from China, supplied corticosterone. Affbiotech supplied primary antibodies for NF-κB p65, NF-κB p-p65 (Ser536) and synaptophysin, with GSDMD (Full length + N terminal), ASC and NLRP3 antibodies being provided by abclonal. Proteintech delivered antibodies specific to PSD95, keap1, IL-1β, and β-tubulin. Abmart supplied Anti-HO-1 and Nrf2. Huabiotech supplied cleaved-caspase1, p-Nrf2, AKT, p-AKT (S473), PI3K, p-PI3K and Lamin B1 antibodies. Zenbio supplied NQO1 antibody. To evaluate cell viability, the cell counting kit-8 was obtained from NCMbiotech in China. Beyotime provided the ROS detection kit, utilizing DCFH-DA for intracellular H_2_O_2_ and oxidative stress analysis. The FAM-FLICA Caspase Assay kit was bought from Immunochemistry Technologies.

## Cell culture and treatment

The differentiated PC12 cell cultures were cultivated in DMEM supplemented with 10% FBS, and incubated in a 5% CO2 atmosphere at 37°C. Once the cells achieved approximately 80% confluence, they were transferred to 6-well plates for further culturing. After a serum-free overnight incubation, the cells underwent treatment with CA and 400 μM corticosterone for a period of 24 h.

### Cell viability assay

PC12 cell viability was evaluated using the MTT as per the manufacturer’s instructions. The cells were seeded at a concentration of 5,000 cells per well in 96-well dishes and grown in Dulbecco’s Modified Eagle Medium (DMEM), with three wells per experimental group. After the treatment, 10 μL of the MTT solution were added to every well, followed by an incubation at 37°C for four more hours. Discard the supernatant and add 150 μL of DMSO to each well. Afterward, the level of light absorption was measured at a wavelength of 490 nm.

### RNA sequencing

Set up three groups: the control group (con group), the corticosterone group (CORT group), and the CA group. The con group was incubated with serum-free DMEM medium, the CORT group with 400 μM corticosterone, and the CA group with both 200 μM CA and 400 μM CORT. After 24 h, PC12 cells were collected from each group (n = 3 for each group), and libraries were constructed and sequenced by Wuhan Maiwei Metabolic Biotechnology Co., Ltd. DESeq2 software was used to analyze differential gene expression, with criteria of *p*-value ≤ 0.05 and | Log2 Fold Change| ≥ 1. To conduct KEGG pathway analysis and Gene Ontology (GO) functional enrichment, visit the Metascape website at https://metascape.org.

### ROS measurements

The levels of intracellular hydrogen peroxide and oxidative stress were evaluated using the DCFH-DA assay. PC12 cells were seeded at a density of 5 × 10 ^4 cells per well in 24-well plates and treated with either regular medium or 400 μM corticosterone for 24 h, with or without CA at 100 μM or 200 μM concentrations. Following this, the DCFH-DA assay was applied to measure changes in ROS levels, adhering to the guidelines provided by the supplier. Cells were exposed to DCFH-DA at 37°C for half an hour, then the dye was eliminated and the cells were washed two times with PBS at a pH of 7.4. Analysis was performed using a Leica fluorescence microscope from Germany and ImageJ software version 1.49 from the NIH in the United States.

## Determination of SOD, LDH and MDA

MDA levels, SOD activity, and LDH content were assessed with assay kits from Nanjing Jiancheng Bioengineering Institute, in accordance with the manufacturer’s instructions.

## Molecular docking

Keap1’s crystal structure was obtained from the RCSB Protein Data Bank website. Modifications, including the elimination and hydrogenation of ethanol and water molecules, as well as the refinement of amino acids, were performed using AutoDock 1.5.7. The 3D chemical structure of CA was obtained from PubChem, its energy was minimized, and the outcome was stored in MOL2 format. This structure was then imported into AutoDock 1.5.7, where all flexible bonds were set to rotate by default and the docking ligands were prepared in PDBQT format. Docking was executed with AutoDock Vina 1.1.2, and the results were visualized using PyMOL.

### Cellular thermal shift assay

The Cell Thermal Shift Assay (CETSA) was utilized to evaluate the stability of the Keap1 protein following the previous protocol (Qin et al., 2022). PC12 cells were cultured in a dish with a density of 1 × 10 ^ 7 cells per dish. Cells were cultured in DMEM medium containing or lacking 200 μM CA for a period of 2 h. After the incubation period, the cells were collected, rinsed with PBS, and then suspended in 500 μL of PBS. The cells were then divided into seven aliquots. These samples were heated at temperatures ranging from 42°C to 54°C in increments of 2°C for 3 min each. Then, the samples experienced alternating rounds of being frozen in liquid nitrogen and then thawed in a water bath at 25°C, this process was done three times. After centrifuging each sample at 12,000 revolutions per minute for 20 min, the liquid above the sediment was moved to a small tube made by Eppendorf. Subsequently, Western blot analysis was performed using a Keap1-specific antibody.

### Extraction of proteins followed by analysis using Western blotting

Cellular proteins were extracted from each sample using a pre-heated buffer containing sodium dodecyl sulfate (SDS) (100 μL). The nuclear protein extraction kit (Solarbio, China) was employed to extract nuclear proteins from cells. 10 μL samples of cell lysates or nuclear were separated on 8%–10% SDS-polyacrylamide gels and then transferred to PVDF membranes from Millipore. The membranes were blocked using 5% nonfat milk in TBST buffer for 60 min at room temperature, then incubated with primary antibodies overnight at 4°C. After triple washing with TBST, the membranes were further incubated with suitable secondary antibodies (Bioprimacy, China) for 60 min at room temperature (The dilutions of antibodies used in the western blotting assay are listed in Table 1). Protein bands were identified utilizing an ECL system and recorded with an imaging device from Bio-Rad in the United States. The intensity of the bands was measured using ImageJ software, and the visualization of the data was done using GraphPad Prism 8. The presented data are pooled from a minimum of three separate experiments.

**TABLE 1:** Antibodies used in this study. Abbreviations used are as follows: r-rabbit, m-mouse, p-phosphorylated, WB-western blot.

Antibody	Host	WB Dilution	Secondary antibody dilution
PSD95	r	1:1500	1:8000
Synaptophysin	r	1:1000	1:8000
NLRP3	r	1:1000	1:8000
Caspase1 p10	r	1:500	1:8000
ASC	r	1:1000	1:8000
IL-1β	m	1:500	1:8000
GSDMD	r	1:1000	1:8000
Nrf2	r	1:2000	1:8000
p-Nrf2	r	1:1000	1:8000
HO-1	r	1:1000	1:8000
NQO1	r	1:1500	1:8000
NF-κB p65	r	1:2000	1:8000
NF-κB p-p65	r	1:1000	1:8000
Keap1	m	1:1500	1:8000
β-tubulin	m	1:8000	1:8000
Lamin B	r	1:4000	1:8000
β-actin	r	1:5000	1:8000

### Flow cytometry assay

After treating with pancreatic enzymes, PC12 cells were collected and then centrifuged at 1000 g for 5 min. Post-centrifugation, the cells underwent a PBS rinse for two times before being resuspended. For the staining process, the FAM-FLICA Caspase Assay kit was employed. Initially, the cells were treated with a diluted Caspase-1 probe for approximately 30 min, which was then followed by a brief incubation with a diluted PI stain for 5 min. The cellular analysis was subsequently carried out on a flow cytometer to assess the staining outcomes. The total rate of pyroptosis is calculated as the percentage of activated Caspase1-positive cells.

### Statistical analysis

For comparing two groups in the CCK-8 assay, a Welch’s *t*-test was applied. Group comparisons were examined through one-way ANOVA, with subsequent analysis using the Least Significant Difference (LSD) test. Results are expressed as the mean ± SEM and are derived from three separate experimental trials. A significance level of p < 0.05 was taken into account for statistical analysis.

## Results

### CA protects PC12 cells against neurotoxicity induced by CORT

**FIGURE 1:**
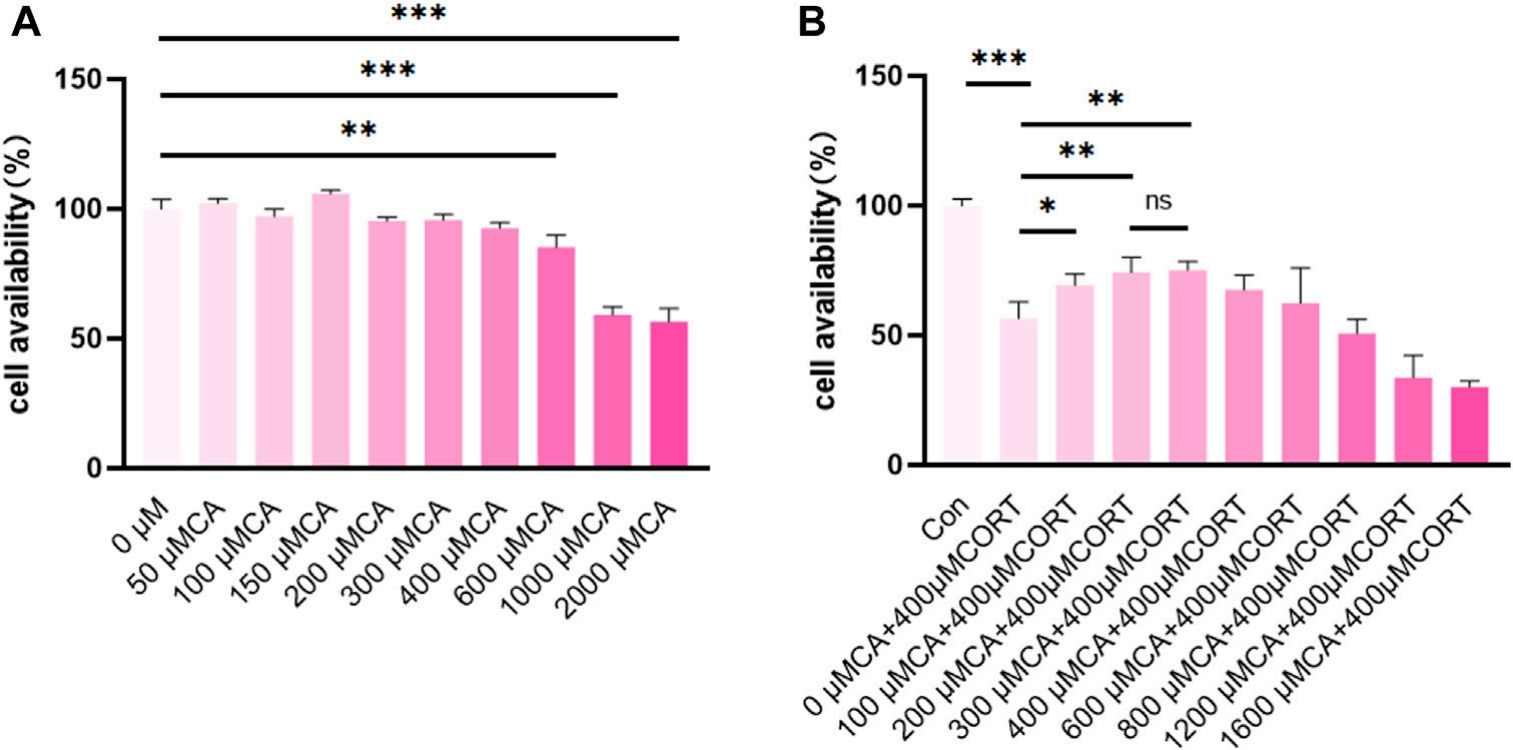
CA protects PC12 cells from CORT-induced neurotoxicity. **(A, B)** cell availability; Significant difference *p < 0.05, **p < 0.01,***p < *0.001*. Data are mean ± SEM (n = 3).

As shown in Figures 1A, B, the viability of PC12 cells significantly decreased at CA concentrations exceeding 600 μM. Additionally, CA at concentrations of 100 μM, 200 μM, and 300 μM enhance the viability of PC12 cells when exposed to 400 μM CORT. However, no significant differences were observed in the impact of 200 μM and 300 μM CA on cells exposed to CORT. Therefore, concentrations of 100 μM and 200 μM CA were chosen for further experiments.

### CA mitigates CORT-induced synaptic impairment in PC12 cells

**FIGURE 2:**
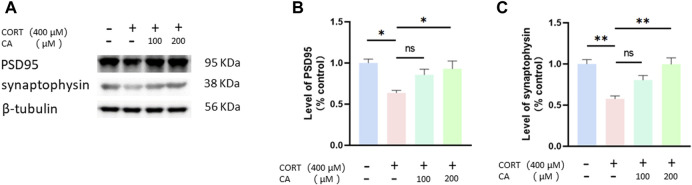
CA increases PSD95 and synaptophysin protein in PC12 cells. **(A)** Representative blot. **(B, C)** semiquantitative analysis of PSD95 and synaptophysin. Significant difference *p < 0.05, **p < *0.01, ns = not significant*. Data are mean ± SEM (n = 3).

Figure 2A–C illustrate that CORT reduces the expression of PSD95 and synaptophysin proteins in PC12 cells after a 24-hour incubation. Conversely, co-treatment with 200 μM CA alongside CORT significantly mitigates this effect.

### Mechanism prediction and experimental verification of CA protection against CORT-induced damage in PC12 cells

**FIGURE 3:**
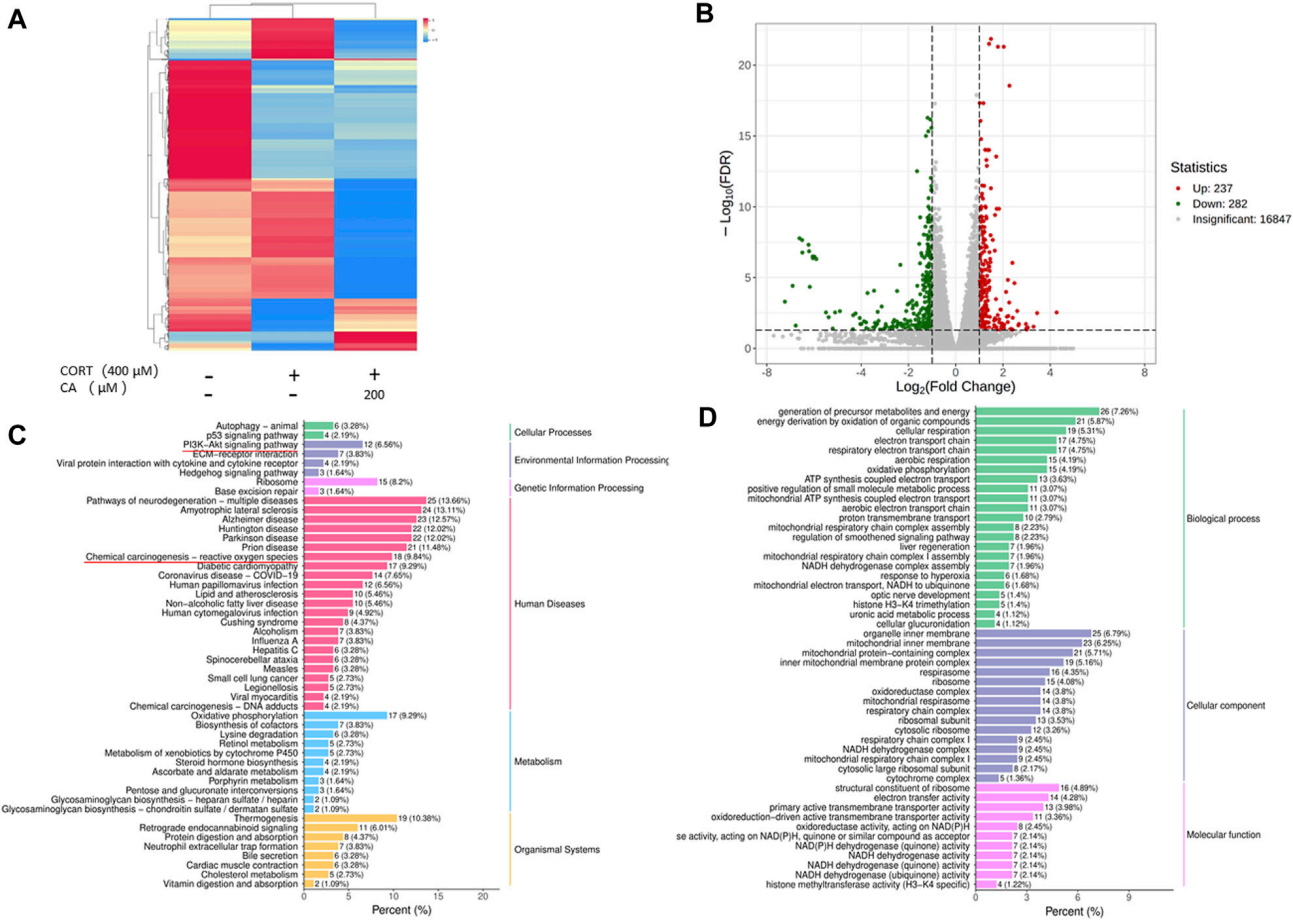
Mechanism prediction of CA protection against CORT-induced damage in PC12 cells by transcriptomics. **(A)** Hitmap of differential gene expression. **(B)** Volcano plot analysis (CORT vs.200 μM CA). **(C)** KEGG enrichment analyses of differential genes (CORT vs.200 μM CA). **(D)** GO enrichment analyses of differential genes (CORT vs.200 μM CA).

PC12 cells were subjected to three conditions: untreated, exposed to 400 μM CORT alone, and co-treated with 400 μM CORT and 200 μM CA for 24 h RNA-seq transcriptome analysis was then conducted. As shown in Figure 3A, CA reversed some of the differential gene expression induced by CORT. The CA group exhibited differential expression in 519 genes, with 237 genes upregulated and 282 genes downregulated, compared to the CORT group (Figure 3B). Analysis of enriched GO terms and KEGG pathways in genes differentially expressed between the 200 μM CA group and the CORT group indicates that CA plays a potential role in protecting cells from ROS-induced damage in neurological disorders. This protection occurs mainly through the modulation of energy metabolism-related biological processes and cellular components, and the PI3K/AKT signaling pathway. (Figures 3C, D). The results suggest that CA may protect cells from CORT-induced damage by activating the PI3K/AKT pathway and reducing oxidative stress.

### CA alleviates CORT-induced damage in PC12 cells by reducing the generation of ROS and MDA, while also enhancing SOD activity

**FIGURE 4:**
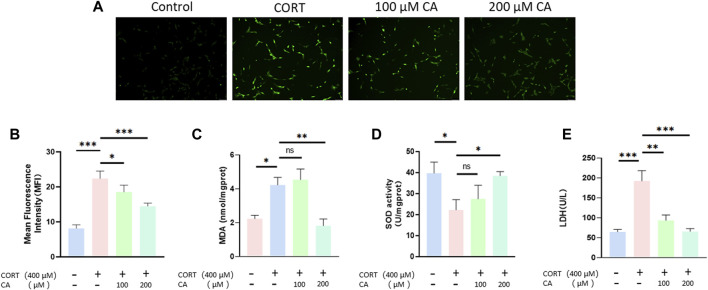
Effect of CA on oxygen species (ROS) production, extracellular LDH, intracellular MDA levels and SOD activity in PC12 cells. **(A)** Representative fluorescence images of ROS production in PC12 cells. **(B)** Intracellular ROS level of PC12 cells. **(C)** Intracellular MDA levels of PC12 cells **(D)** Intracellular SOD activity in PC12 cells. **(E)** Extracellular LDH levels of PC12 cells. Significant difference *p < 0.05, **p < 0.01,***p < *0.001*. Data are mean ± SEM (n = 3).

Figure 4A, B illustrate that the presence of 100 μM and 200 μM CA significantly decreases ROS production in PC12 cells. Intracellular levels of MDA and SOD activity exhibited distinct responses to treatment (Figures 4C, D). Specifically, MDA levels increased and SOD activity decreased after incubation with 400 μM CORT. In contrast, treatment with 200 μM CA ameliorated these effects. Consequently, CA reduces CORT-induced damage in PC12 cells, resulting in decreased LDH release, as depicted in Figure 4E.

### CA reduced CORT-induced pyroptosis in PC12 cells

**FIGURE 5:**
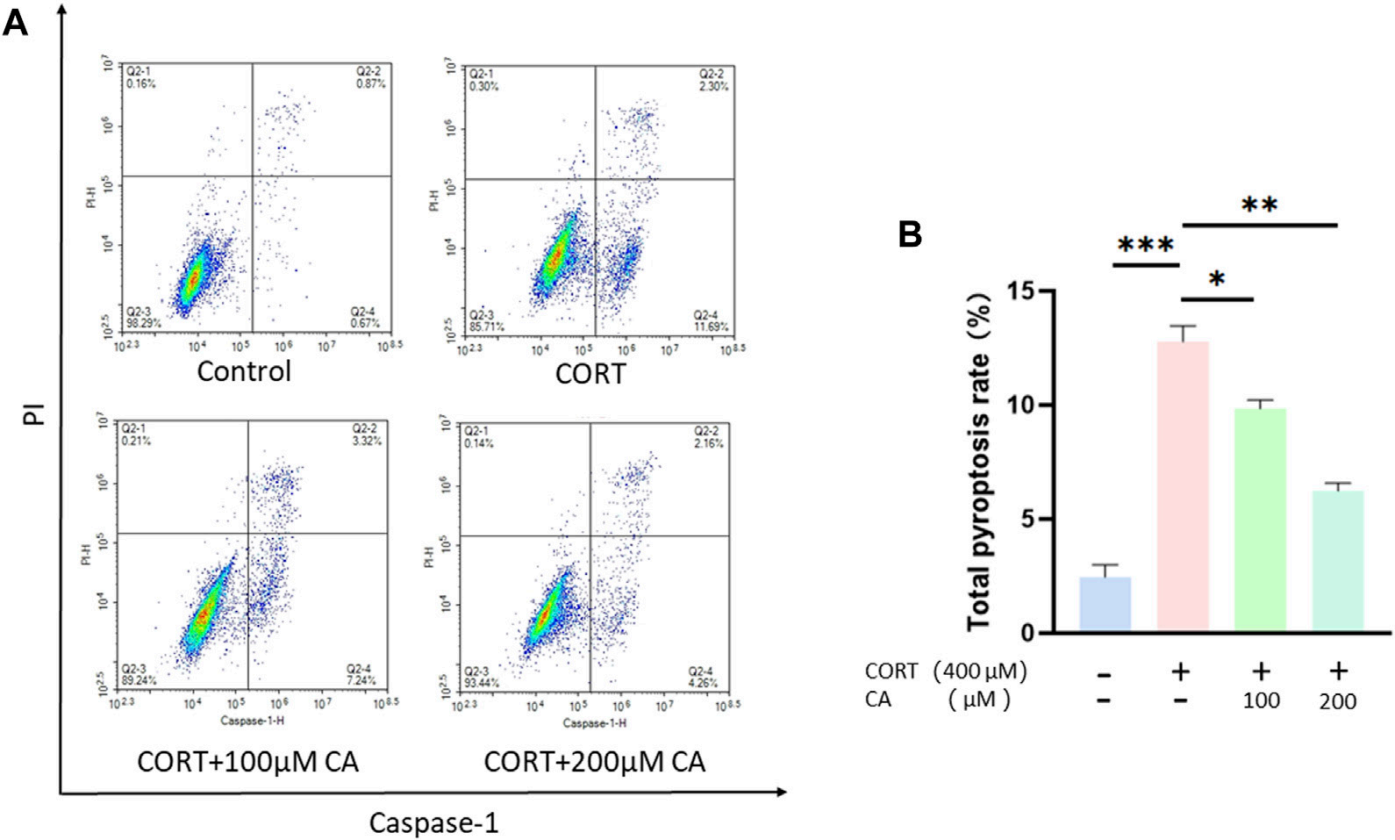
CA reduce pyroptosis rate in PC12 cells. **(A)** Representative images of flow cytometry. **(B)** Pyroptosis rate in PC12 cells. Significant difference *p < 0.05, **p < 0.01,***p < *0.001*. Data are mean ± SEM (n = 3).

**FIGURE 6:**
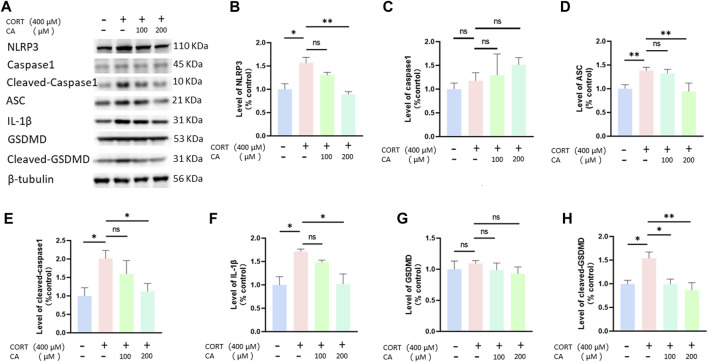
CA effects pyroptosis-associated proteins in PC12 cells. **(A)** Representative blot. **(B–H)** semiquantitative analysis of NLRP3, caspase1, ASC, cleaved-caspase1, IL-1β, GSDMD and Cleaved-GSDMD. Significant difference *p < 0.05, **p < *0.01, ns = not significant*. Data are mean ± SEM (n = 3).

Previous research indicates that CORT can increase ROS generation, thus inducing pyroptosis in nerve cells. To investigate whether CA could protect PC12 cells from CORT-induced pyroptosis, we employed a flow cytometry assay. As illustrated in Figure 5, incubation with CORT alone resulted in a total pyroptosis rate exceeding 12% in PC12 cells. Co-incubation with 100 μM and 200 μM CA significantly alleviated this effect. We consequently examined the changes in pyroptosis-associated proteins in PC12 cells using western blotting assay. Figure 6 illustrates a notable increase in the levels of NLRP3, ASC, cleaved-caspase1, IL-1β, and cleaved-GSDMD following CORT treatment, while CA mitigates this impact.

### CA activates CORT-induced inhibition of the PI3K/AKT signal pathway

**FIGURE 7:**
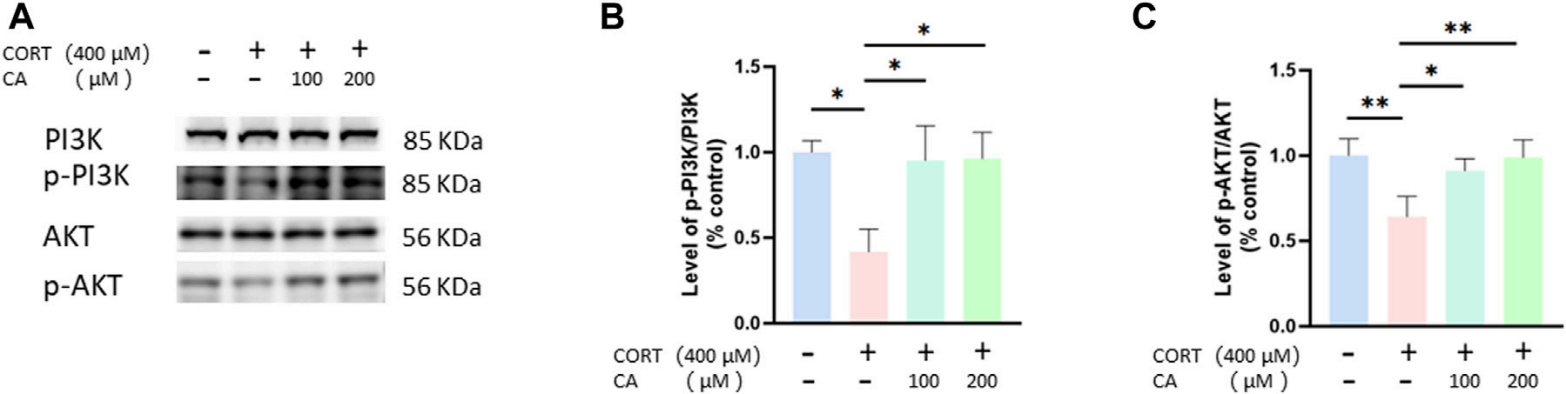
CA influence PI3K/AKT signal pathway in PC12 cells. **(A)** Representative blot. **(B, C)** semiquantitative analysis of p-PI3K/PI3K and p-AKT/AKT. Significant difference *p < 0.05, **p < *0.01, ns = not significant*. Data are mean ± SEM (n = 3).

To empirically confirm the transcriptomic findings, we conducted Western blot assays to assess the PI3K/AKT pathway in PC12 cells. Figure 7 illustrates that treatment with 200 μM CA markedly elevated p-PI3K and p-AKT levels, which were diminished in the CORT-exposed group. Based on these results, we suggest that 200 μM CA may reactivate the PI3K/AKT signal pathway, previously inhibited by CORT, potentially offering a protective benefit.

### CA stimulates the Nrf2/ho-1/NF-κB pathway in PC12 cells

**FIGURE 8:**
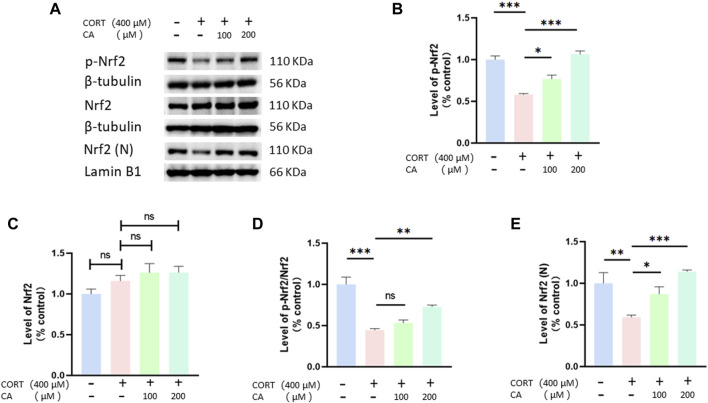
CA influence Nrf2, p-Nrf2 and Nrf2 (N) in PC12 cells. **(A)** Representative blot. **(B–E)** semiquantitave analysis of p-Nrf2, Nrf2 p-Nrf2/Nrf2 and Nrf2 (N). Significant difference *p < 0.05, **p < 0.01, ***p < 0.001, ns = not significant. Data are mean ± SEM (n = 3)

**FIGURE 9:**
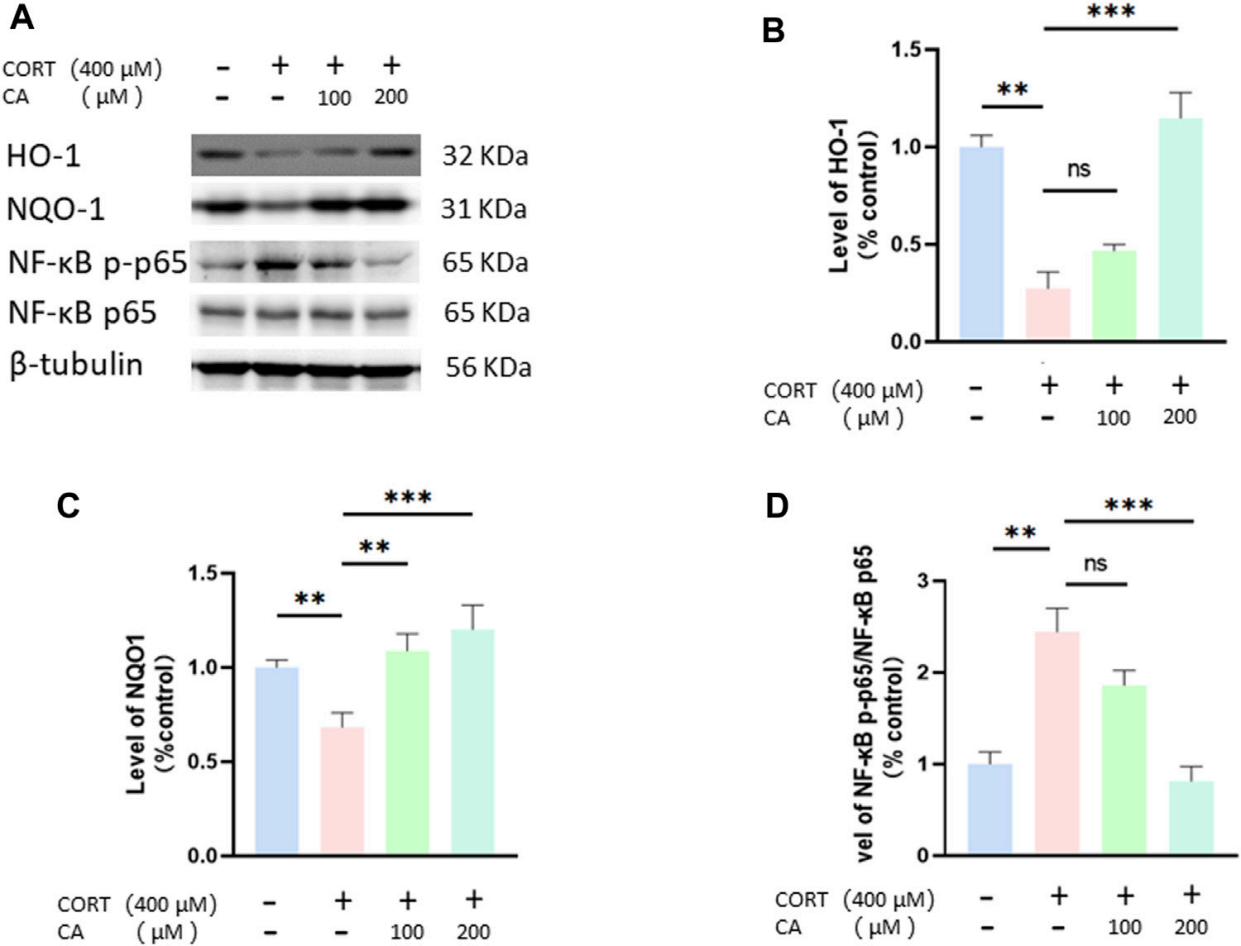
CA influences HO-1, NQO1 and NF-κB in PC12 cells. **(A)** Representative blot. **(B–D)** semiquantitative analysis of p-Nrf2, HO-1, NQO1 and NF-κB p-p65. Significant difference *p < 0.05, **p < 0.01,***p < *0.001, ns = not significant*. Data are mean ± SEM (n = 3).

To further elucidate how CA protects PC12 cells from CORT-induced effects, we employed Western blotting assay. As depicted in Figures 8, 9, CORT significantly decreases the levels of HO-1, NQO1 and p-Nrf2 and Nrf2 in the nucleus (Nrf2 (N)), while simultaneously increasing the level of phosphorylated NF-κB p-p65. Conversely, the levels of overall Nrf2 and NF-κB remains unchanged. The test showed that 200 μM CA significantly boosted the levels of p-Nrf2, Ho-1 and NQO1, while reducing the levels of NF-κB p-p65 compared to the control groups.

### CA can bind to Keap1 protein, as revealed by molecular docking and CETSA

**FIGURE 10:**
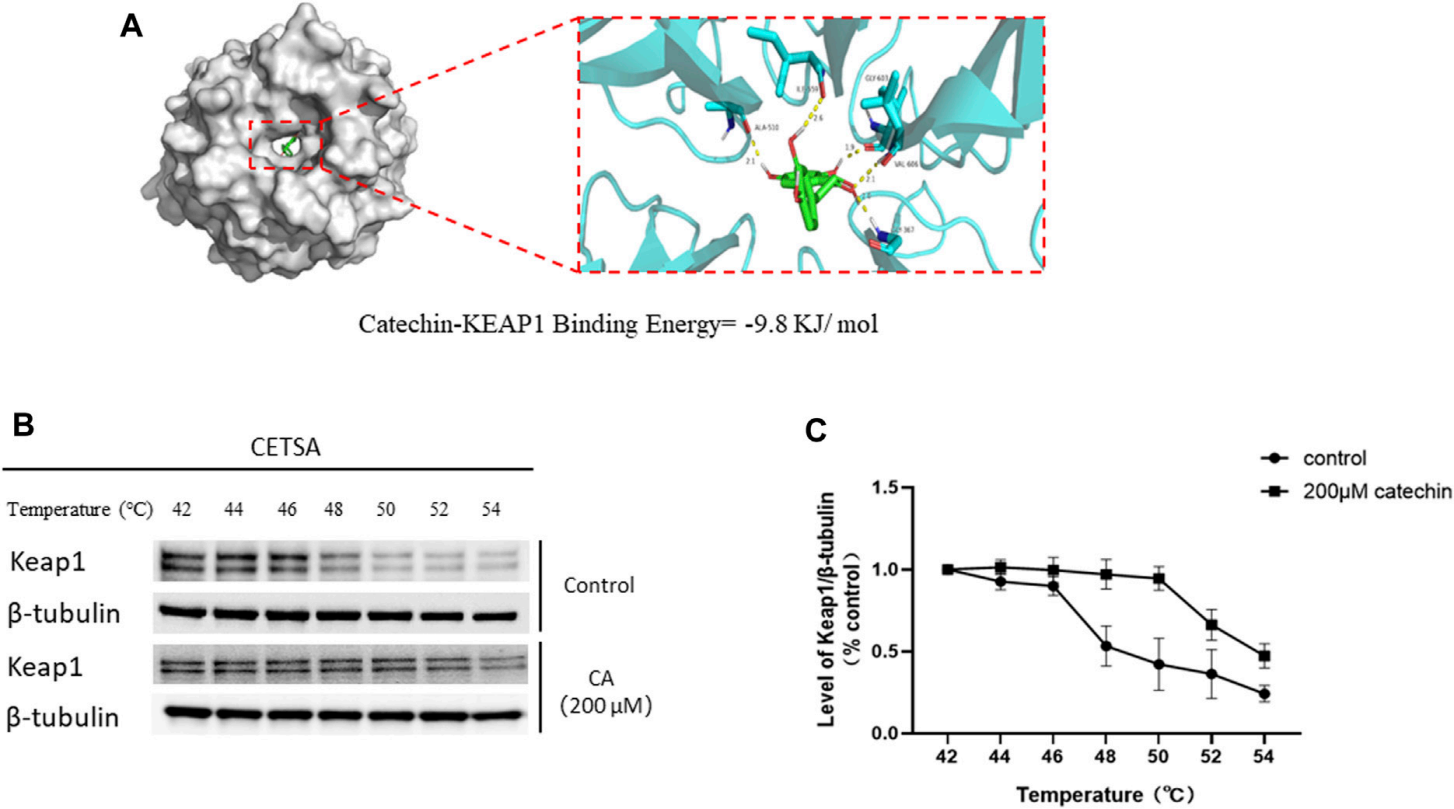
Molecular docking and thermal stability test of CA and Keap1 protein based on PC12 cells. **(A)** Molecular models of Catechin binding to Keap1 proteins. **(B)** Representative blot in Cellular thermal shift assay (CETSA). **(C)** Quantification of Keap1 abundance from CETSA.

As shown in Figure 10A, CA binds to the Keap1 protein with a binding energy of −9.8 kJ/mol, indicates a strong binding affinity. In the PC12-based CETSA, we assessed the thermal stability of Keap1 using a specific Keap1 antibody. The results indicated that the thermal denaturation profile of Keap1 became significantly more stable upon incubation with CA (Figures 10B, C).

## Discussion

It is widely acknowledged that prolonged exposure to stress can lead to depression. When a stressor is detected, the HPA axis becomes more excitable, leading to the release of glucocorticoids from the adrenal glands (Muscari et al., 2022). Among these, cortisol,a glucocorticoid, is found at abnormally high levels in both the plasma and brain tissue of individuals suffering from depression (Blanco and Conant, 2021). Previous *in vitro* studies have demonstrated that cortisol can inflict harm on PC12 cells, establishing the cortisol-induced PC12 cell model as a widely accepted *in vitro* representation of depressive conditions (He et al., 2023). In this research, we uses PC12 cells and 400 μM CORT to simulate a cellular model of depression following previous study (Zhou et al., 2017). Our cell viability assays revealed that CA at concentrations below 400 μM do not adversely impact the survival of PC12 cells. Furthermore, when PC12 cells are co-treated with varying concentrations of CA alongside a fixed 400 μM concentration of CORT, compared to 0 μM of CA, we observed enhanced cell viability at the 100 μM, 200 μM, and 300 μM levels of CA. Interestingly, the viability of PC12 cells showed no notable variation when exposed to 200 μM and 300 μM doses of CA in combination with CORT. Consequently, we selected the concentrations of 100 μM and 200 μM for CA to conduct our subsequent experiments.

Neuroplasticity refers to the brain’s ability to undergo temporary changes in its organization and function in response to different stimuli, such as stress. This adaptability enables the brain to adjust and reorganize itself to accommodate environmental changes. Recent research has established a strong connection between depressive disorders and hippocampal synaptic plasticity (Parekh et al., 2022). Synaptophysin and PSD95 are proteins associated with the presynaptic and postsynaptic elements (Lai et al., 2022). Studies have shown that increasing the levels of synaptophysin and PSD95 in the hippocampus of mice can help alleviate depression-like behaviors (Kong et al., 2023). Our experimental findings indicate that exposure to CORT diminished the expression of synaptophysin and PSD95 in PC12 cells. In contrast, concurrent treatment with 200 μM CA and CORT enhances the expression of these proteins. This suggests that CA may protect PC12 cells from CORT-induced synaptic dysfunction.

To investigate the potential mechanisms of underlying CA’s protective effect on cell damage induced by CORT, we collected PC12 cells from three groups (control group, CORT group, and 200 μM CA group) and conducted RNA-seq transcriptome analysis. The protective effect of CA on CORT-induced damage in PC12 cells is hypothesized to stem from its capacity to regulate intracellular functions, modulate the PI3K/AKT signaling, and reduce ROS production.

Oxidative stress occurs when the body’s oxidation and antioxidant systems are imbalanced due to external factors, resulting in the excessive production of ROS (Zhang B. et al., 2022; Zorov et al., 2014). ROS, MDA and SOD are key indicators for assessing oxidative stress (Zhou et al., 2017; Chao et al., 2021). Excessive ROS levels can cause lipid peroxidation, resulting in the creation of MDA and the linking of large molecules like proteins and nucleic acids, which can harm cell wellbeing. The concentration of MDA is a significant marker that indicates the body’s antioxidant potential, offering insight into the rate and severity of lipid peroxidation, and thereby indicating the extent of oxidative tissue damage (Veljovic et al., 2022). SOD, an antioxidant enzyme present in living organisms, facilitates the conversion of superoxide anion radicals into oxygen and hydrogen peroxide. This enzyme plays a crucial role in balancing oxidative and antioxidant processes in the body, and is closely associated with the development and progression of depressive disorders (Jiménez-Fernández et al., 2022). Moreover, LDH is widely present in animals, plants, microorganisms, and isolated cells, acting as the final rate-limiting enzyme in glycolysis and catalyzing the reversible reaction between pyruvate and lactate. When cells are damaged and rupture, LDH leaks into the extracellular space, making it an important indicator for assessing cell damage. Our study demonstrates that CA at concentrations of 100 μM and 200 μM can reduce the elevated extracellular LDH levels in the PC12 cell model of depression. This suggests that CA provides protection to PC12 cells in the depressive model, aligning with the findings of the cell viability assays. Furthermore, compared to the CORT group, the 200 μM group shows a significant decrease in ROS and MDA levels and a marked increase in SOD levels. This suggests that CA exerts its protective effect on PC12 cells damaged by CORT by mitigating oxidative stress and consistent with the predictions of transcriptomics.

Pyroptosis, a caspase1-dependent form of cell death, is regulated by GSDM family proteins, such as GSDMD. This process is characterized by inflammasome activation, cell membrane pore formation, and the release of IL-1β (Shi et al., 2015; Kuang et al., 2017). The NLRP3 inflammasome is central to mediating pyroptosis, exerting a critical influence within the pathway. Studies indicate that excessive ROS production, causing oxidative stress, plays a key role in triggering the NLRP3 inflammasome, which is associated with various neurological disorders, such as depression (Zhong et al., 2018; Han et al., 2021; Tao et al., 2021; Gu et al., 2022). Consequently, interventions aimed at mitigating oxidative stress, ROS generation, NLRP3 inflammasome activity and pyroptosis are emerging as crucial targets for depression therapy. Previous research has found that CORT incubation can induce pyroptosis in PC12 cells (Chai et al., 2022). Therefore, we examined the pyroptosis in PC12 cells using flow cytometry and detected the expression of pyroptosis-related proteins through Western blot analysis. The findings suggest that CA can reverse CORT-induced pyroptosis in PC12 cells. The effect may be attributed to the anti-oxidative stress properties of CA.

We investigated whether CA’s ability to reduce CORT-induced oxidative stress aligns with transcriptomics predictions. Western blot analysis confirmed that CA activates the PI3K/AKT signaling pathway, findings consistent with our transcriptomic results. Moreover, the PI3K/AKT signaling pathway has been documented to influence neural plasticity and cell viability, a basically conclusion that aligns with our observations regarding synaptic plasticity and cell viability (Na et al., 2022). Nrf2 is recognized as a crucial regulatory factor that maintains the body’s redox balance by functioning as a transcription factor in response to oxidative stress (Ma, 2013). Keap1 acts as a suppressor of Nrf2, when bound, Nrf2 is rendered inactive. However, the dissociation of Keap1 from Nrf2 leads to Nrf2 phosphorylation and subsequent nuclear translocation. Within the nucleus, Nrf2 bines to antioxidant response elements, triggering the transcription of genes pivotal for antioxidant defense, including Ho-1, NQO1, and SOD (Liao et al., 2023). Additionally, research has found that the activation of Nrf2 and HO-1 can suppress NF-kB activation, thereby inhibiting NLRP3 and reducing cell pyroptosis (Zhang Y. et al., 2022). The PI3K/AKT pathway acts as an upstream signal to Nrf2, and studies have demonstrated that activation of the PI3K signaling pathway can enhance the Nrf2 phosphorylation and nuclear translocation (Chou et al., 2023; Hannan et al., 2020). This perspective supports our research findings on the PI3K/AKT signaling pathway and Nrf2, p-Nrf2, as well as Nrf2 (N). Moreover, previous studies have shown that CA can induce the dissociation of Keap1 and Nrf2 by forming a stable bond with the Nrf2 protein on Keap1, leading to the activation of Nrf2 (Islam et al., 2023; Jing et al., 2018). Therefore, molecular docking studies were performed revealing that CA forms a stable complex with the Keap1 protein. This was further substantiated by cellular thermal shift assays, which demonstrated that CA enhances Keap1 stability at elevated temperature. Hence, we propose two mechanisms by which CA activates Nrf2 to reduce ROS generation and prevent cell pyroptosis: one involves activating the PI3K/AKT signaling pathway, and the other entails binding with Keap1.

However, this study exclusively examined the potential protective effects of CA and its underlying molecular mechanisms using a cellular model of depression, rather than an animal model, which introduces certain limitations to the research. To determine whether CA possesses potential for development as an antidepressant drug, *in vivo* experiments are imperative. Despite these limitations, our research provides valuable reference points for future studies.

## Conclusion

**FIGURE 11:**
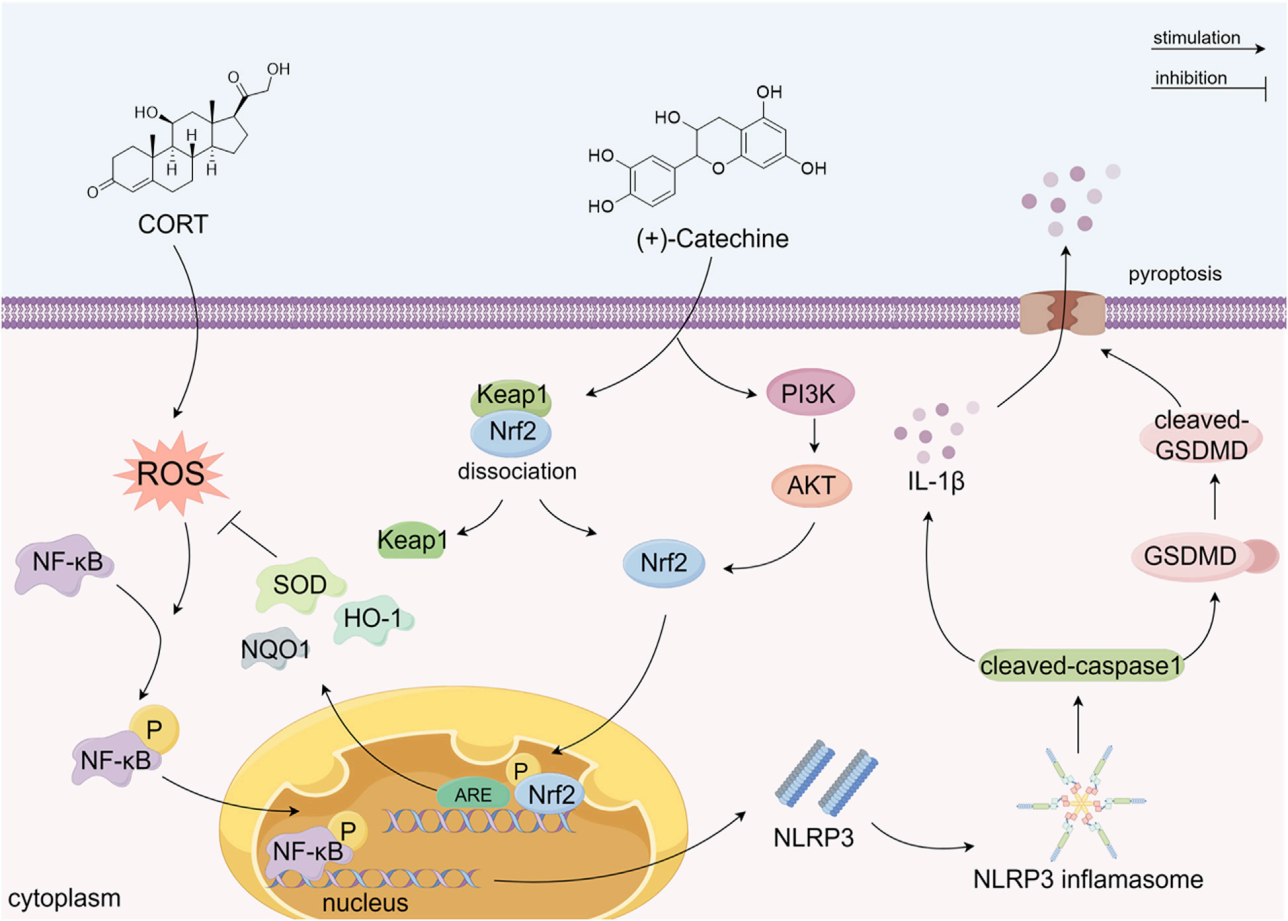
The possible mechanism of CA alleviating CORT-induced damage in PC12 cells. (By Figdraw).

In summary, our study explored the protective effects of CA on cell depression models induced by CORT and its underlying mechanisms. The results confirmed that CA plays a significant protective role against CORT-induced cell damage. As shown in Figure 11, this protective effect is believed to stem from CA’s activation of the PI3K/AKT signaling pathway and its interaction with Keap1, which leads to Nrf2 phosphorylation and subsequent activation of the Nrf2/HO1 pathway. This activation reduces ROS production and inhibits NF-κB phosphorylation. It is believed that these processes collectively suppress NLRP3 formation and the expression of pyroptosis-related proteins. The study highlights the potential of CA in alleviating oxidative stress and pyroptosis in the CORT-induced cell depression model, suggesting a promising avenue for the development of innovative antidepressant treatments.

## Declaration of Generative Al and AI-assisted technologies in the writing process

In the preparation of this manuscript, the authors have utilized ChatGPT 4.0 for polishing the language, but have not employed AI for the acquisition of scientific perspectives. All language, after being polished, has been reviewed by the authors. The authors assume full responsibility for the content of the publication.

## Data Availability

The data presented in the study are deposited in the NCBI repository, accession number PRJNA1148779. Available at: https://www.ncbi.nlm.nih.gov/bioproject/PRJNA1148779.
